# Language learning, language use and the evolution of linguistic variation

**DOI:** 10.1098/rstb.2016.0051

**Published:** 2017-01-05

**Authors:** Kenny Smith, Amy Perfors, Olga Fehér, Anna Samara, Kate Swoboda, Elizabeth Wonnacott

**Affiliations:** 1 University of Edinburgh, Edinburgh, UK; 2 University of Adelaide, Adelaide, Australia; 3 University College London, London, UK

**Keywords:** learning, iterated learning, language

## Abstract

Linguistic universals arise from the interaction between the processes of language learning and language use. A test case for the relationship between these factors is linguistic variation, which tends to be conditioned on linguistic or sociolinguistic criteria. How can we explain the scarcity of unpredictable variation in natural language, and to what extent is this property of language a straightforward reflection of biases in statistical learning? We review three strands of experimental work exploring these questions, and introduce a Bayesian model of the learning and transmission of linguistic variation along with a closely matched artificial language learning experiment with adult participants. Our results show that while the biases of language learners can potentially play a role in shaping linguistic systems, the relationship between biases of learners and the structure of languages is not straightforward. Weak biases can have strong effects on language structure as they accumulate over repeated transmission. But the opposite can also be true: strong biases can have weak or no effects. Furthermore, the use of language during interaction can reshape linguistic systems. Combining data and insights from studies of learning, transmission and use is therefore essential if we are to understand how biases in statistical learning interact with language transmission and language use to shape the structural properties of language.

This article is part of the themed issue ‘New frontiers for statistical learning in the cognitive sciences’.

## 1. Introduction

Natural languages do not differ arbitrarily, but are constrained so that certain properties recur across languages. These linguistic universals range from fundamental design features shared by all human languages to probabilistic typological tendencies. Why do we see these commonalities? One widespread intuition (see e.g. [1]) is that linguistic features which are easier to learn or which offer advantages in processing and/or communicative utility should spread at the expense of less learnable or functional alternatives. They should therefore be over-represented cross-linguistically, suggesting that linguistic universals arise from the interaction between the processes of language learning and language use.

In this paper, we take linguistic variation as a test case for exploring this relationship between language universals and language learning and use. Variation is ubiquitous in languages: phonetic, morphological, syntactic, semantic and lexical variation are all common. However, this variation tends to be predictable: usage of alternate forms is conditioned (deterministically or probabilistically) in accordance with phonological, semantic, pragmatic or sociolinguistic criteria. For instance, in many varieties of English, the last sound in words like ‘cat’, ‘bat’ and ‘hat’ has two possible realizations: either [t], an alveolar stop, or [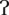], a glottal stop. However, whether [t] or [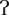] is used is not random, but conditioned on linguistic and social factors. For instance, Stuart-Smith [2] showed that T-glottaling in Glaswegian varies according to linguistic context, style, social class of the speaker and age of the speaker ([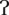] is most frequent before a pause, and less frequent at a pauseless word boundary; glottaling is more common in more informal speech, working-class speakers T-glottal more than middle-class speakers, with pre-pausal glottaling being essentially obligatory for working-class speakers, and younger speakers T-glottal more frequently than older speakers, with the glottal being obligatory in a wider range of contexts). Similar patterns of conditioned variation are found in morphology and syntax. Truly free variation, where there are no conditioning factors governing which variant is deployed in which context, is rare or entirely absent from natural language [3].

How can we explain the conditioned nature of variation in natural language? Does this property of natural language reflect biases in language learning, or are there other factors at play? In this paper, we review three strands of experimental work exploring these questions, and introduce new modelling and experimental data. We find that while the biases of language learners can potentially play a role in shaping this feature of linguistic systems, the relationship between biases of learners and the structure of languages is not straightforward.

The structure of the paper is as follows. Section 2 reviews the literature showing that language learners (children in particular) are biased against learning linguistic systems exhibiting unpredictable variation, and tend to reduce or eliminate that variation during learning. This suggests a straightforward link between their learning biases and the absence of unpredictable variation in language. However, in §3 we present computational modelling and empirical results showing that weak biases in learning can be amplified as language is passed from person to person. This means that we can expect to see strong effects in language (e.g. the absence of unconditioned variation) even when learners do not have strong biases. Transmission of language in populations can also produce the opposite effect, *masking* the biases of learners: a population's language might retain variability even though every learner is biased against acquiring such variation. In the final section (§4), we show that pressures acting during language use (the way people adjust their language output in order to be understood, or the tendency to reuse recently heard forms) may also shape linguistic systems. This means that caution should be exercised when trying to infer linguistic universals from the biases of learners, or vice versa, because the dynamics of transmission and use, which mediate between learner biases and language design, are complex.

## 2. Learning

In a pioneering series of experiments, Hudson Kam & Newport [4,5] used statistical learning paradigms with artificial languages to explore how children (age 6 years) and adults respond to linguistic input containing unpredictably variable elements. After a multi-day training procedure, participants were asked to produce descriptions in the artificial language, the measure of interest being whether they veridically reproduced the ‘unnatural’ unpredictable variation in their input (a phenomenon known as ‘probability matching’), or reduced/eliminated that variability. Their primary finding was that children tend to regularize, eliminating all but one of the variants during learning, whereas adults were more likely to reproduce the unconditioned variation in their input. This difference between the learning biases of adults and children suggests that the absence of unpredictable variation in human languages may be a consequence of biases in child language acquisition.

It remains an open question why children might have stronger biases against unpredictable variation than adults. A common hypothesis is that children's bias toward regularization might be due to their limited memory [4–6]. However, these accounts are hard to reconcile with other research indicating that limitations of this type do not necessarily lead to more regularization [7,8]; while it is possible that memory limitations may play a role, it seems unlikely that they are the main driving force behind this behaviour. Consistent with this, there is evidence that learners bring domain-specific biases to the language learning task: experimental paradigms which compare this tendency to regularize in closely matched linguistic and non-linguistic tasks indicate stronger biases for regularity in language [9,10], suggesting that learners may expect language or communicative conventions more generally not to exhibit unpredictable variation (a point we return to in §4).

The biases of learners also interact with features of the linguistic input. For instance, adults tend to regularize more when the input is both unpredictable and complex (e.g. when there are multiple unpredictably varying synonymous forms) but can acquire quite complex systems of conditioned variation (e.g. where there are multiple synonymous forms whose use is lexically or syntactically conditioned: [5,11]). There is also suggestive evidence that conditioning facilitates the learning of variability by children, although they are less adept at acquiring conditioned variation than adults [11,12]. Similarly, if the learning task is simplified by mixing novel function words and grammatical structures with familiar English vocabulary, children's tendency to regularize is reduced [13].

Some types of linguistic variability are also more prone to regularization than others. Culbertson *et al*. [14] show that adult learners given input exhibiting variable word order will favour orders where modifiers appear consistently before or after the head of a phrase; children show a similar pattern of effects, with a stronger bias [15]. Finally, the assumptions learners make about their input also affect regularization: adults are far more likely to regularize when unconditioned input is ‘explained away’ as errors by the speaker generating that data [16].

Taken together, these various factors suggest that regularization of inconsistent input cannot be explained solely as a result of learner biases. The nature of those biases, the complexity of the input learners receive, and the pragmatic assumptions the learner brings to the task all shape how learners respond to linguistic variation. Importantly, regularization is not an all-or-nothing phenomenon—the strength of learners' tendency to regularize away unpredictable variation can be modulated by domain, task difficulty and task framing. Furthermore, rather than being categorically different in their response to variation, adults and children appear to have biases that differ quantitatively rather than qualitatively. Adults might regularize more given the right input or task framing, and children will regularize less given the right kind of input. This suggests that, while rapid regularization driven by strong biases in child learning may play a role in explaining the constrained nature of variation in natural language, there is a need for a mechanistic account explaining how weaker biases at the individual level could have strong effects at the level of languages.

## 3. Transmission

### (a) Iterated learning and regularization

As well as being restructured by the biases of individual language learners, languages are shaped by processes of transmission. Modelling work exploring how socially learned systems change as they are transmitted from person to person has established that weak biases in learning can be amplified as a result of transmission (e.g. [17]): their effects accumulate generation after generation. The same insight has been applied to the regularization of unpredictable variation. Reali & Griffiths [18] and Smith & Wonnacott [19] use an experimental iterated learning paradigm where an artificial language is transmitted from participant to participant, each learner learning from data produced by the previous participant in a chain of transmission. Both studies show that unpredictable variation, present in the language presented to the first participant in each chain of transmission, is gradually eliminated, resulting in the emergence of languages entirely lacking unpredictable variation. This happens even though each learner has only weak biases against variability—both studies used adult participants and relatively simple learning tasks, providing ideal circumstances for probability matching.

**Figure 1.**
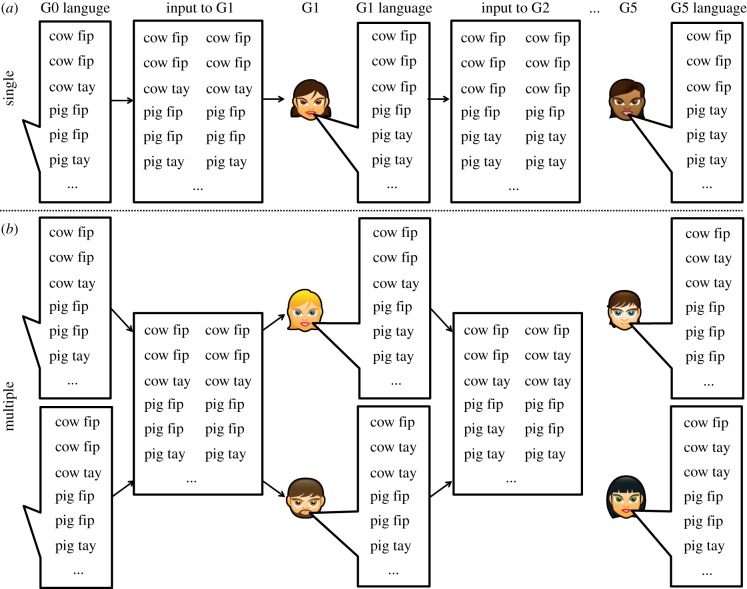
Illustration of single-person and multiple-person chains, here with *S* = 2. In single-person chains (*a*), each individual learns from the (duplicated, as *S* = 2) data produced by the single individual at the previous generation. In multiple-person chains (*b*), each individual learns from the pooled language produced by the individuals at the previous generation, with all individuals at a given generation being exposed to the same pooled input. (Online version in colour.)

Let us consider one of these studies in more detail. Smith & Wonnacott [19] trained participants on a miniature language for describing simple scenes involving moving animals, where every scene consisted of one or two animals (pig, cow, giraffe or rabbit) performing an action (a movement), and the accompanying description consisted of a nonsense verb, a noun, and (for scenes featuring two animals) a post-nominal marker indicating plurality. This plural marker varied unpredictably: sometimes plurality was marked with the marker *fip*, sometimes with the marker *tay*. After training on this miniature language, participants labelled the same scenes repeatedly, generating a new miniature language. The language produced by one participant was then used as the training language for the next participant in a chain of transmission, passing the language from person to person (figure 1*a*).

When trained on an unpredictably variable input language, most participants in this experiment reproduced that variability fairly faithfully: their use of the plural marker was statistically indistinguishable from probability matching. However, when the language was passed from person to person, plural marking became increasingly predictable. While some chains of transmission gradually converged on a system where only one plural marker was used (e.g. plurality was always marked with *fip*, with *tay* dying out), the most common outcome after five ‘generations’ of transmission was a conditioned system of variation: some nouns marked the plural with *fip*, other nouns used *tay* and the choice of marker was entirely predicted by the noun being marked. The language as a whole therefore retained variation, but (as in natural languages) that variability was conditioned, in this case, on the linguistic (lexical) context.

This shows that transmission of language from person to person via iterated learning, intended as an experimental model of how languages are transmitted ‘in the wild’ provides a potential mechanism explaining how weak biases against variability in individuals could nonetheless produce categorical effects in languages. One important implication is that we cannot directly infer learner biases from the features of language: there can be a categorical prohibition on unconditioned variation in language without a corresponding categorical prohibition on acquiring variation at the individual level. Nor can we straightforwardly infer linguistic universals from learner biases: learners can have weak biases, which accumulate over transmission to produce categorical effects in the language.

### (b) Iterated learning in populations

One feature of the experimental method used in Smith & Wonnacott [19] is that each learner learns from the output of one other learner. This is a clear disanalogy with the transmission of natural languages, where we encounter and potentially learn from multiple other individuals. Furthermore, there is reason to suspect that the rapid emergence of conditioned variation in our experiment was at least speeded up by this design decision. The initial participant in each chain was trained on an input language where all nouns exhibited exactly the same frequency of use of the two markers, *fip* and *tay*. These participants produced output that was broadly consistent with their input data in exhibiting variability in plural marking. However, their output was typically not *perfectly* unconditioned—they tended to overproduce one marker with some nouns, and the other marker with other nouns. While we were able to verify that this conditioning was no greater than one would expect by chance for most participants, it nonetheless introduced statistical tendencies, which were identified and exaggerated by the next participant in the chain. In this way, initial ‘errors’ in reproducing the perfectly unpredictable variation gradually snowballed to yield perfectly conditioned systems.

By similar reasoning, one might expect that learning from multiple individuals at each generation might not result in the elimination of variation in the same way. In the experiments with single learners, the ‘errors’ made by the initial participant in each chain were random: one participant might overproduce *fip* for giraffe and pig when attempting to reproduce the perfectly variable language, another might overproduce that marker for pig, rabbit and cow. In single chains, these errors get exaggerated over time. But in multiple-participant chains, because this accidental conditioning would probably differ between participants, combining the output of multiple participants should mask the idiosyncratic conditioning produced by each participant individually. Thus, given a large enough sample of learners, their collective output would look perfectly unconditioned, even if each individual was in fact producing rather conditioned output.

This suggests that in transmission chains where each generation consists of *multiple* participants, each learning from the pooled linguistic output of the entire population at the previous generation (figure 1*b*), the emergence of conditioned variation might not occur at all, or at least might be slower. If true, this would have important implications for the more general issue of how learner biases map on to language structure. In particular, we might expect to see substantial *mismatches* between learner biases and language structure. In the next two subsections, we test this hypothesis using a computational model and an experiment with human participants, both based closely on Smith & Wonnacott [19]; we then return to potential implications for our understanding of the mapping between properties of individuals and properties of language.

### (c) Learning from multiple people: model

Following Reali & Griffiths [18], we model learning as a process of estimating the underlying probability distribution over plural markers, and production as a stochastic process of sampling marker choices given this estimate (for alternative modelling approaches, see [20–22]). More specifically, we treat learning as a process of Bayesian inference: learners observe multiple plural markers, infer the underlying probability distribution over markers via Bayes Rule, and then sample markers according to that probability.

#### (i) Description of the model

We model a scenario based closely on Smith & Wonnacott [19]: learners observe multiple descriptions, each consisting of a noun and a plural marker, where there are 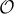 nouns (numbered 1 to 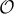) and two possible plural markers, *m*_1_ (e.g. *fip*) and *m*_2_ (e.g. *tay*). For noun *i*, the learner's task is to infer the underlying probability of marker *m*_1_, which we will denote as *θ_i_* (the probability of marker *m*_2_ for noun *i* is 1 − *θ_i_*). If the learner sees *N* plurals for noun *i*, then the likelihood of *n* occurrences of *m*_1_ and *N* − *n* occurrences of *m*_2_ is given by the Bernoulli distribution
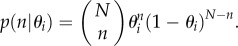
Assuming that the learner infers *θ* independently for each noun, the posterior probability of a particular value of *θ_i_* given *n* occurrences of *m*_1_ with noun *i* is then given by
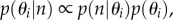
where *p*(*θ*) is the prior probability of *θ*. The prior captures the learner's expectations about the distribution of the two markers for each noun. Following Reali & Griffiths [18], we use a symmetrical Beta distribution with parameter *α* = 0.26 for our prior: when *α* = 0.26 (and in general when *α* < 1) the prior is U-shaped, and learners assume before encountering any data that *θ* will either be low (i.e. *m*_1_ is almost never used to mark the plural) or that *θ* will be high (i.e. the plural is almost always marked with *m*_1_), but that the probability of intermediate values of *θ* (i.e. around 0.5) is low. This prior captures a learner who assumes that plural marking for any given noun will tend not to be variable, although this expectation can be overcome given sufficient data. Reali & Griffiths [18] show that human performance on a task in which learners learn a system of unpredictable object labelling is best captured by a model where *α* = 0.26, i.e. exhibiting a preference for consistent object labelling, justifying our choice of prior favouring regularization.^1^

We can use this model of learning to model iterated learning,^2^ in a similar scenario to that explored experimentally by Smith & Wonnacott [19]—the precise details of the model are taken from the experiment in §3.4. We use a language in which there are three nouns (i.e. 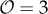; one might imagine that they label three animals: cow, pig and dog). A population consists of a series of discrete generations, where generation *g* learns from data (plural marker choices) produced by generation *g* − 1.

To explore the effects of mixing input data from multiple learners, we model two kinds of population. In *multiple-person* chains, each generation consists of *S* individuals (we consider *S* = 2, 5 or 10). Each individual estimates *θ* for each noun based on their input (i.e. the output of the preceding generation). They then produce *N* = 6 marker choices for each noun, data which form the input to learning at the next generation. In the simplest version of the multiple-person model, we assume each learner observes and learns from the combined output of all *S* individuals in the previous generation—i.e. if they receive input from two individuals, one who uses *m*_1_ twice (and *m*_2_ four times), the other who uses *m*_1_ four times (and *m*_2_ twice), they will estimate *θ* for that noun based on six instances of *m*_1_ and six of *m*_2_.

In *single-person* chains, each generation consists of a single individual, who estimates *θ* for each noun based on the output of the preceding generation, and then produces *N* = 6 marker choices for each noun according to the estimated values of *θ*, which are passed on to the next generation. In order to allow us to directly compare single-person chains with multiple-person chains, and to allow more straightforward comparison to the experimental results presented later, we will initially assume that each learner in a single-person chain observes the data produced by the previous generation *S* times. For instance, if *S* = 2, then in the single-person case, if a learner learns from an individual who produced *m*_1_ twice (and *m*_2_ four times), that learner will estimate *θ* based on data consisting of four occurrences of *m*_1_ and eight of *m*_2_. Duplicating data in this way ensures that, by holding *S* constant while comparing single-person and multiple-person chains, we can explore the effects of learning from multiple individuals without confounding this manipulation with either the total amount of data learners receive or the number of data points each individual produces. These two schemes for iterated learning are illustrated in figure 1. We consider an alternative model of single-person chains (where each learner produces *SN* marker choices for each noun) below.

We construct an initial set of markers (our generation 0 language) where every noun is marked with marker *m*_1_ four times from a total of six occurrences (i.e. *n*/*N* = 4/6, both markers are used for every noun, with *m*_1_ being twice as frequent as *m*_2_). We then run 100 chains of transmission in both single-person and multiple-person conditions, matching every multiple-person chain with a single-person chain with the same value of *S*. Our goal is to measure how the variability of plural marking evolves over time, and specifically whether multiple-person chains show reduced levels of conditioning or regularization relative to single-person chains.

#### (ii) Measures of variability

There are several measures of interest that we can apply to these simulations. Firstly, we can track the overall variability of the languages produced by our simulated population. The simplest way to do this is to track *p*(*m*_1_), the frequency with which plurals are marked using marker *m*_1_, across all nouns and all speakers (*p*(*m*_1_) = 2/3 in the initial language in each chain, and will be approximately 1 or 0 for a highly regular language in which every individual uses a single shared marker across all nouns). Overall variability can also be captured by the *entropy* of marker use across all nouns and all speakers, which we will denote *H*(Marker). We use Shannon entropy, so variability is measured in the number of bits required per marker to encode the sequence of markers produced by a population:
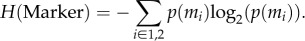
*H*(Marker) will be high (≈1) when both markers are used equally frequently and the language is maximally variable, and zero when only one marker (*m*_1_ or *m*_2_) is used. Measuring entropy rather than *p*(*m*_1_) allows us to meaningfully average over chains that converge on predictably using one marker, regardless of whether that marker is *m*_1_ or *m*_2_.

We can also measure the conditional entropy of marker use given the noun being marked—this is the measure used by Smith & Wonnacott [19] to quantify the emergence of conditioned variation
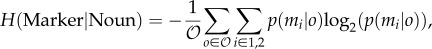
where *p*(*m_i_*|*o*) is the frequency with which plurals for noun *o* are marked using marker *m_i_*, across all speakers.^3^ *H*(Marker|Noun) will be high when the language exhibits variability and that variability, aggregating across individuals, is unconditioned on the noun being marked. Low *H*(MarkerJNoun) indicates either the absence of variability or the conditioning of variation, such that each noun is usually/always marked with a particular plural marker (e.g. some nouns might reliably appear with *m*_1_, others with *m*_2_).

Finally, we can measure the extent to which variability is conditioned on both the noun being marked and the identity of the speaker, calculated as
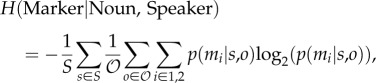
where 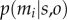 is the frequency with which plurals for noun *o* are marked using marker *m_i_* by speaker *s*. High *H*(Marker|Noun, Speaker) indicates variation which is unconditioned by linguistic context or speaker identity (i.e. truly free variation); low *H*(Marker|Noun, Speaker) indicates a system where each speaker exhibits a (potentially idiosyncratic) conditioned system of variation.

Table 1 gives examples of these various measures of variability.

**Table 1.** Measures of variability for illustrative scenarios in a population consisting of a single speaker (s1: first three rows) or two speakers (s1 and s2, remaining rows), each speaker producing two labels for each of two nouns (cow and pig) using two plural markers (*fip* and *tay*). Note that, for a language produced by a single speaker, *H*(Marker|Noun) and *H*(Marker|Noun, Speaker) are necessarily identical.

language	*P*(*fip*)	*H*(Marker)	*H*(Marker|Noun)	*H*(Marker|Noun, Speaker)
s1: cow fip, cow fip, pig fip, pig fip	1	0	0	0
s1: cow fip, cow fip, pig tay, pig tay	0.5	1	0	0
s1: cow fip, cow tay, pig fip, pig tay	0.5	1	1	1
s1: cow fip, cow fip, pig fip, pig fip	1	0	0	0
s2: cow fip, cow fip, pig fip, pig fip				
s1: cow fip, cow fip, pig tay, pig tay	0.5	1	0	0
s2: cow fip, cow fip, pig tay, pig tay				
s1: cow fip, cow fip, pig tay, pig tay	0.5	1	1	0
s2: cow tay, cow tay, pig fip, pig fip				
s1: cow fip, cow tay, pig fip, pig tay	0.5	1	1	1
s2: cow fip, cow tay, pig fip, pig tay				

#### (iii) Results: learning from multiple speakers slows regularization and conditioning of variation

**Figure 2.**
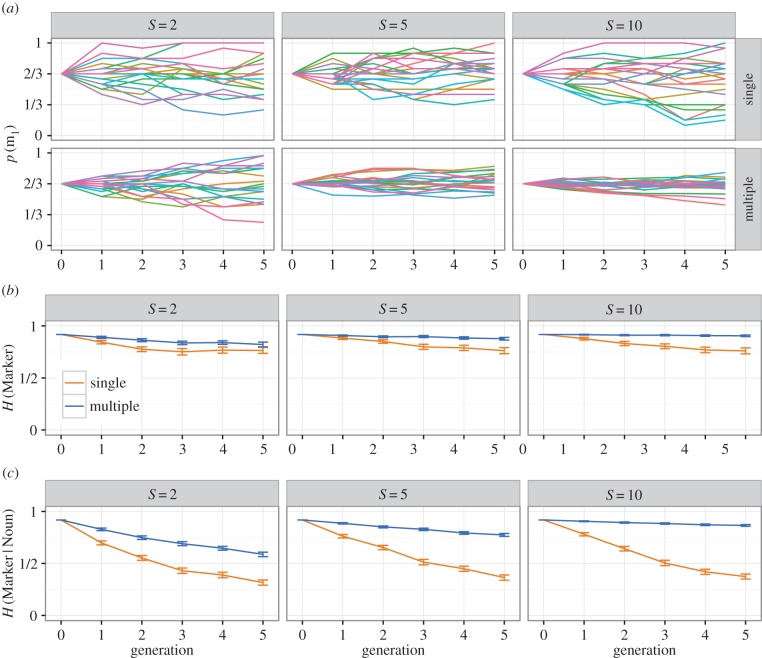
Simulation results. (*a*) Proportion of plurals marked using *m*_1_. Each line shows an individual chain, for 20 simulation runs. In single-person chains, we see greater divergence between chains, with some chains converging on always or seldom using *m*_1_; by contrast, in multiple-person chains, particularly for larger *S*, the initial level of variability is retained longer. (*b*) Entropy of plural marking, averaged over 100 runs, error bars indicate 95% CIs. This overall measure of variability shows the trend visible in (*a*) more clearly: while variability is gradually lost over generations in all conditions (as indicated by reducing *H*(Marker)), this loss of variability is slower in multiple-person chains, particularly with larger *S*. (*c*) Conditional entropy of plural marking given the noun being marked, averaged over the same 100 runs. While we reliably see the emergence of conditioned variation in single-person chains, as indicated by reducing *H*(Marker|Noun), this process is slowed in multiple-person chains, particularly for larger *S*.

Figure 2 shows how the frequency of use of the two markers evolves over five simulated generations of transmission. In the single-person chains, individual chains diverge in their usage of the singular marker, with some chains overusing *m*_1_ and other chains underusing it. In multiple-person chains, mixing of the productions of multiple individuals slows this divergence, and with *S* = 10 the initial proportion of marker use is reasonably well preserved even after five generations.

These same tendencies can be seen more clearly in the plots of entropy (figure 2*b*): entropy decreases over time in both conditions, but more slowly for multiple-person chains, particularly with high *S*. Finally, *H*(Marker|Noun) declines in all conditions, reflecting the gradual emergence of lexically conditioned systems of variation similar to those seen in Smith & Wonnacott [19], but this decrease in conditional entropy is slower in multiple-person chains, as predicted. *H*(Marker|Noun, Speaker) (not shown) shows a very similar pattern of results: because they learn from a shared set of data, each speaker's usage broadly mirrors that of the population as a whole.

#### (iv) Extension: an alternative model of single-person populations

The same pattern of results holds if we compare multiple-person chains to single-individual chains where the single individual produces *SN* times for each noun, rather than producing *N* times and then duplicating this data *S* times. This alternative means of matching single- and multiple-person chains removes the potential under-representativeness of the small sample of data produced in the single-person chains, which arises from producing *N* data points and then duplicating: duplication will produce datasets that provide a relatively noisy reflection of the speaker's underlying *θ*, because a smaller sample is always less representative, while (due to duplication) masking this noisiness for the learner. In the revised model where each learner produces *SN* times for each noun, single-person chains *still* regularize faster than multiple-person chains. This is because the outcome in each chain is more influenced by misestimations of *θ* by single learners; as the learners have a regularization bias, these mis-estimations are more likely to be towards regularity. In multiple-person chains, these misestimations have a smaller effect, because the input each learner receives is shaped by the *θ* estimates of multiple individuals who generate their data. We focus on the duplication-based version of the single-person model because it more closely matches our experimental method, both from Smith & Wonnacott [19] and in the new experimental data presented below: in experiments with human participants, requiring participants in single-person chains to produce more data than participants in multiple-person chains is potentially problematic, because participants might be expected to become more regular in later productions (due to fatigue, boredom, or priming from their previous productions).

#### (v) Extension: tracking speaker identity

One feature of our multiple-person chains is that each learner learns from the aggregated input of the entire previous generation, and attempts to estimate a single value of *θ* for each noun to account for the data produced by the entire population. An alternative possibility is that learners entertain the possibility that different speakers contributing to their input have different underlying linguistic systems (in our case, different values of *θ*). Burkett & Griffiths [23] provide a general framework for exploring Bayesian iterated learning in populations where learners entertain the possibility that their input consists of data drawn from multiple distinct languages, and infer the distribution of languages in their input. Here, we adopt a slightly different approach, and assume that learners can directly exploit social cues during learning.

There is a wealth of evidence from natural language that linguistic variation can be conditioned on various facets of speaker identity. For example, there is a large literature demonstrating differences in male and female language use (e.g. [24–27]): speakers associate certain variants with gender and avoid variants they perceive as gender-inappropriate [28,29]. We can capture this kind of speaker-based conditioning in our model by assuming that learners attempt to estimate a value of *θ* for each individual speaker contributing to their input, and then produce variants according to their estimate of *θ* for the speakers in their input who match them according to sociolinguistic factors (e.g. being of the same gender, similar social class and so on). This potentially opens up a wide range of hierarchical learning models in which speakers integrate data from multiple sources according to sociolinguistically mediated weighting factors.

We consider only the simplest such model here, which matches the experimental manipulation in the next section and which constitutes the logical extreme of this kind of identity-based conditioning of variation: we assume that every individual at generation *g* is matched in some way to a single individual in generation *g* − 1 (e.g. according to some combination of gender, ethnicity, age, social class and so on). The learner then estimates *θ* based on their matched individual's behaviour. For instance, if the individuals in each generation are numbered from 1 to *S*, individual *s* in generation *g* estimates *θ* for each noun based on the data produced by individual *s* at generation *g* − 1. We will refer to this as the ‘speaker identity’ variant of the model, and contrast it with the ‘no speaker identity’ model outlined in the preceding sections, where learners simply combine data from multiple speakers without attending to the identity of the speakers who produced it. These two models therefore lie at extremes of a continuum from unweighted combination of data from multiple speakers (the no speaker identity model) to the most tightly constrained use of sociolinguistic factors (the speaker identity model)—models in which learners integrate input from multiple individuals in a more sophisticated manner will lie somewhere between these two extremes.

**Figure 3.**
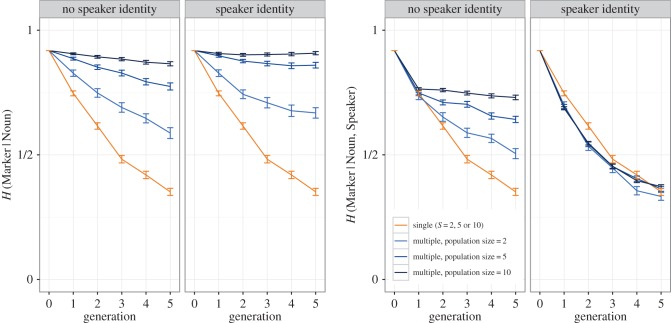
Simulation results for multiple-person chains, where we manipulate whether learners can access and exploit speaker identity during learning. Results for single-person chains are shown for reference, all results are averaged over 100 simulation runs. The pair of plots on the left shows *H*(Marker|Noun), i.e. conditional entropy of plural marking given the noun being marked, averaged across the population—this captures the extent to which there is a conditioned system of variability that is shared and therefore independent of speaker. There is no effect of the speaker identity manipulation here, and learning from multiple people slows the development of a population-wide system of conditioned variation. The right pair of plots shows *H*(Marker|Noun, Speaker), i.e. conditional entropy of plural marking, given the noun being marked *and* the speaker—this measure captures the development of speaker-specific systems of conditioned variation, where each speaker conditions their marker use on the noun being marked, but allows that different speakers might use different systems of conditioning. Here, we see an effect of the speaker identity manipulation: in the no speaker identity models, these results essentially mirror those for *H*(Marker|Noun), i.e. no conditioning develops due to mixing of data from multiple speakers; however, in the model where learners can track speaker identity, speaker-specific systems of conditioning emerge, as indicated by smoothly reducing *H*(Marker|Noun, Speaker).

Figure 3 shows the results for these two variants of the multiple-person model, for *H*(Marker|Noun) (a measure which aggregates across the entire population), and for *H*(Marker|Noun, Speaker) (a measure which captures speaker-specific conditioning). In the basic variant of the model where learners do not attend to speaker identity, these two measures show similar trends: while each individual speaker is somewhat more predictable than the population mean, learning from multiple speakers still slows the conditioning of variation, as each learner is blind to these idiosyncratic individual consistencies. By contrast, when learners attend to speaker identity and base their behaviour on data from a single individual at the previous generation, the slowing effect of learning from multiple teachers disappears: because each individual is only tracking the behaviour of a single individual at the previous generation, each population essentially consists of *S* individual chains, each regularizing independently; the population's collective language regularizes only slowly because individual chains of transmission are independent and can align only by chance, but the individual chains show the same rapid conditioning of variation we see in single-person chains.

Weaker variants of the speaker identity model (e.g. where learners combine input from multiple speakers in a way that is weighted by the social identity of those speakers and their own social identity) should produce results that tend towards the results seen in the no-identity models as the strictness of social conditioning diminishes (that is, they should show slowed regularization and conditioning). Where human learners lie on this continuum is an empirical question, which we touch upon in the next section—at this point we simply note that the contrast between *H*(Marker|Noun) and *H*(Marker|Noun, Speaker) serves as a diagnostic of whether or not learners attend to speaker identity when learning and reproducing variable systems. More generally, this difference in the behaviour of the model based on speaker identity shows that the cumulative regularization effect is not only modulated by the number of individuals a learner learns from, but also how they handle that input (do they track variant use by individuals, or simply for the whole population?) and how they derive their own output behaviour from their input (do they model a single individual from their input, or produce output which reflects the usage across all their input?).

The model therefore shows that regularization of variation does not automatically ensue from learning and transmission, even in situations where learners have biases in favour of regularity. Smith & Wonnacott [19] showed that weak biases at the individual level can accumulate and therefore be unmasked by iterated learning; this model shows that the reverse is also possible, and that biases in learning can be masked by the dynamics of transmission in populations. Note that this is true even if learners have much stronger biases for regularity than that we used here (see endnote 1). In other words, the slowing effects of learning from multiple speakers apply even if individual learners make large reductions to the variability of their input (as child learners might: [4,5]). We return to the implications of this point in the general discussion.

### (d) Learning from multiple people: experiment

The model outlined in the previous section makes two predictions. First, learning from multiple individuals will slow the cumulative conditioning seen in Smith & Wonnacott [19]. Second, the degree of slowing will be modulated by the extent to which learners are able to attend to (or choose to attend to) speaker identity when tracking variability. We test these predictions with human learners, using a paradigm based closely on Smith & Wonnacott [19].

#### (i) Methods

*Participants*. 150 native English speakers (112 female, 38 male, mean age 21 years) were recruited from the University of Edinburgh's Student and Graduate Employment Service and via emails to undergraduate students. Participants were paid £3 for their participation, which took approximately 20 min.

*Procedure.* Participants worked through a computer program, which presented and tested them on a semi-artificial language. The language was text-based: participants observed objects and text displayed on the monitor and entered their responses using the keyboard. Participants progressed through a three-stage training and testing regime:

1) *Noun familiarization*. Participants viewed pictures of three cartoon animals (cow, pig and dog) along with English nouns (e.g. ‘cow’). Each presentation lasted 2 s, after which the text disappeared and participants were instructed to retype that text. Participants then viewed each picture a second time, without accompanying text, and were asked to provide the appropriate label.
2) *Sentence training*. Participants were exposed to sentences paired with pictures. Pictures showed either single animals or pairs of animals (of the same type) performing a ‘move’ action, depicted graphically using an arrow. Sentences were presented in the same manner as nouns (participants viewed a picture plus text, then retyped the text); each sentence was contained in a speech bubble, and in some conditions an alien character was also present, with the speech bubble coming from their mouth (see below). Each of the six scenes was presented 12 times (12 training blocks, each block containing one presentation of each scene, order randomized within blocks).
3) *Testing.* Participants viewed the same six scenes without accompanying text and were prompted to enter ‘the appropriate description’, where the text they provided appeared contained in a speech bubble; in some conditions, there was also an alien figure present, with the speech bubble coming from their mouth. Each of the six scenes was presented six times during testing (six blocks, order randomized within blocks).

The first participant in each transmission chain was trained on a language in which every sentence consisted of a verb (always *glim*, meaning ‘move’), an English noun (*cow*, *pig* or *dog*), and then (for scenes involving two animals) a plural marker, either *fip* or *tay*. For instance, a scene with a single dog would be labelled *glim dog*, a scene with two cows could be labelled either *glim cow fip* or *glim cow tay*. The critical feature of the input language was the usage of *fip* and *tay*. One marker was twice as frequent as the other: half of the chains were initialized with a language where two-thirds of plurals were marked with *fip* and one third were marked with *tay*, and the other half were initialized with one third *fip*, two-thirds *tay*. Importantly, these statistics also applied to each noun: each noun was paired with the more frequent plural marker eight times and the less frequent marker four times during training. Plural marking in the input language is thus unpredictable: while one marker is more prevalent, both markers occur with all nouns.

We ran the experiment in two conditions, manipulating the number of participants in each generation. Our one-person condition was a replication of [19] with a different participant pool, which also provides a baseline for comparison with our two-person condition. In the one-person condition, 50 participants were organized into 10 diffusion chains. The initial participant in each chain was trained on the input language specified above and each subsequent individual in a chain was trained on the language produced during testing by the preceding participant in that chain.^4^ Each test block from participant *g* was duplicated to generate two training blocks for participant *g* + 1, equivalent to an *S* = 2 single-person chain in the model.

In the two-person condition, each generation in the chain consisted of two participants (not necessarily in the laboratory at the same time), who learned from the same input language. We then combined the descriptions they produced during testing to form a new input language for the next generation. We assigned 100 participants to this condition, organized into 10 diffusion chains. The initial pair of participants in each chain was trained on the input language specified above and each subsequent pair was trained on the language produced during testing by the preceding pair in that chain. That language was created by taking the six blocks of test output from the two participants at generation *g* and combining them (order randomized) to produce 12 blocks of training material for generation *g* + 1 (equivalent to the multiple-person chains with *S* = 2 in the model).

As an additional manipulation in the two-person condition, for half of the chains we provided the identity of the speaker producing each description. Rather than simply presenting descriptions in a dislocated speech bubble, we also included a picture of one of two aliens (who had distinct shapes and colours, and appeared consistently on different sides of the screen): six blocks were presented as being produced by alien 1, six by alien 2. For these *speaker identity* chains, at the testing phase participants were asked to provide the label produced by one of the two aliens (one participant in a pair would produce descriptions for alien 1, one for alien 2). The next generation would then see the data marked with this speaker identity (training blocks produced by participant 1 would appear as being produced by alien 1, training blocks produced by participant 2 would appear as being produced by alien 2), potentially allowing participants to track inter-speaker variability and intra-speaker consistency. In the no speaker identity chains, the alien figures were simply not present: participants saw descriptions in a dislocated speech bubble and typed their own descriptions into the same dislocated bubble, meaning that participants were unable to access or exploit information about how variation was distributed across speakers.

This procedure in the speaker identity chains, where participants observe input from multiple individuals but are prompted to produce as one individual, mirrors the speaker identity version of the multiple-person model, in that each learner is paired with one of multiple speakers who provide their input. In the model, we assumed that learners make maximal use of speaker identity when available, and produce data that match their estimate of their focal input model. Whether or not human learners behave in this way is an empirical question. *If* our experimental participants exploit this social cue in the same way, our model predicts that we should see substantial differences between two-person chains where speaker identity is provided and those where it is not: the latter should show slowed regularization/conditioning of variation, the former should pattern with one-person chains in showing rapid reduction in unpredictable variation. By contrast, if we see *no* difference between variants of the two-person chains (i.e. if both speaker identity and no identity chains show reduced rates of regularization relative to one-person chains), this would suggest that human learners in these conditions do not readily exploit speaker identity information, at least not in an experimental paradigm like this one.

#### (ii) Results

**Figure 4.**
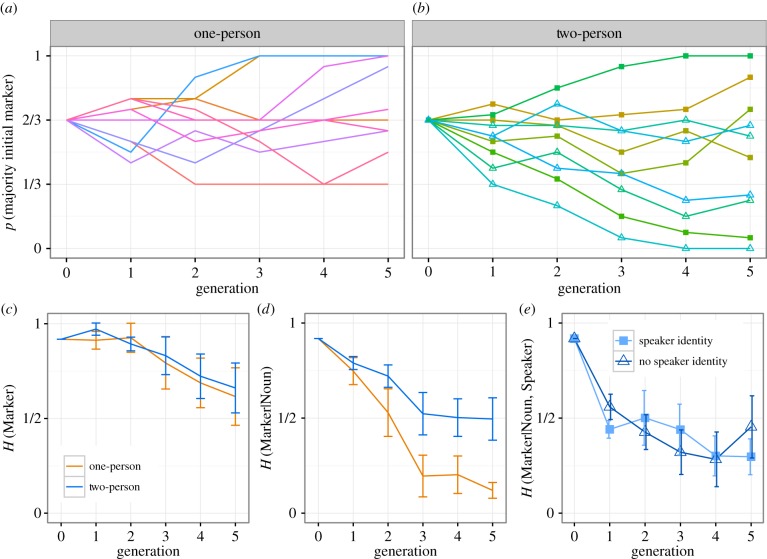
Experimental data. (*a,b*) Proportion of plurals marked using the marker that was initially in the majority in each chain. Each line shows an individual chain. For two-person chains, filled shapes indicate speaker ID provided, hollow shapes indicate no speaker ID. (*c*) Total entropy of the languages, as indicated by *H*(Marker), averaged over all chains (error bars indicate 95% CIs). Overall variability declines only slowly in both conditions: while some chains converge on always or never marking plurals with the majority initial marker, most retain variability in plural marking. (*d*) *H*(Marker|Noun), i.e. conditional entropy of marker choice given noun. In one-person chains, conditioned systems of variability rapidly emerge, as indicated by reducing *H*(Marker|Noun); as predicted by the model, this development of conditioned variation is slowed in two-person chains. (*e*) *H*(Marker|Noun, Speaker), i.e. conditional entropy of marker choice given the noun being marked and the speaker, for two-person chains only, split according to whether participants were provided with information on speaker identity. Contrary to the predictions of the model, providing speaker identity makes no difference to the development of speaker-specific conditioning of variation.

Figure 4*a,b* shows the number of plurals marked with the chain-initial majority marker (i.e. *fip* for chains initialized with two-thirds *fip*). As seen in Smith & Wonnacott [19], while some chains converge on a single marker, most chains still exhibit variation in plural marking by generation 5. A logit regression, collapsing across the speaker identity manipulation^5^ (including condition (sum-coded) and generation as fixed effects, with by-chain random slopes for generation; the same fixed- and random-effect structure was used for all analyses reported here), indicates no effect of generation or condition on majority marker use (generation: *β* = 0.042, *SE* = 0.099, *p* = 0.668; condition: *β* = 0.090, *SE* = 0.106, *p* = 0.392) and a marginal interaction between condition and generation (*β* = −0.199, *SE* = 0.099, *p* = 0.044) reflecting a tendency for two-person chains to underuse the initial majority marker at later generations.

Figure 4*c* shows *H*(Marker) (i.e. overall entropy, calculated over the combined output of both participants in the two-person chains). While entropy is generally high, it declines over generations in both conditions as some chains converge on using a single marker. A linear mixed-effects regression reveals a significant effect of generation on entropy (*β* = −0.063, *SE* = 0.021, *p* = 0.007) with no effect of condition and no interaction between generation and condition, *p* > 0.870.^6^

Figure 4*d* shows *H*(Marker|Noun), i.e. conditional entropy of marker use given the marked noun (calculated over the combined output of both participants in the two-person chains). Collapsing across the speaker identity manipulation^7^, a regression analysis shows a significant effect of generation (*β* = −0.130, *SE* = 0.017, *p* < 0.001) and a significant interaction between generation and condition (*β* = −0.040, *SE* = 0.017, *p* = 0.027), with conditional entropy decreasing more rapidly in one-person chains: as predicted by the models presented in the previous section, mixing of data from multiple individuals reduces the speed with which variation becomes conditioned.

Finally, figure 4*e* shows *H*(Marker|Noun, Speaker) for the two-person chains, i.e. the conditional entropy of marker use given noun and speaker. Providing or withholding speaker identity clearly has no effect on the extent to which speaker-specific conditioning of variation develops, as confirmed by a regression analysis (speaker identity (sum-coded) and generation as fixed effects, with by-chain random slopes for generation), which shows the expected effect of generation but no effect of speaker identity (*β* = 0.006, *SE* = 0.048, *p* = 0.136) and no interaction between generation and speaker identity (*β* = −0.005, *SE* = 0.023, *p* = 0.838). Recall that the multiple-person model presented in the previous section predicted a substantial difference between learners who tracked and exploited speaker identity and those who did not (with slowed regularization/conditioning in the latter case, and results closely matched to the single-person chains in the former case). The absence of a difference in our experimental data therefore suggests that participants were not strongly predisposed to attend to speaker identity during learning; they behaved as if they were tracking the use of plural markers across both speakers, and basing their own behaviour on an estimate of marker usage derived from their combined input.^8^

This finding appears to contradict other empirical evidence reviewed previously, showing that natural language learners *can* exploit social conditioning of linguistic variation. One possible explanation for their failure to do so in the current experimental paradigm is simply that participants were not required to attend to speaker identity during training and did not anticipate that speaker identity would become relevant on test (e.g. the participant briefing in the speaker identity condition did not state in advance that they would be required to produce for one of the aliens). The social categories we used (two distinct types of alien) may also be less salient than if we had used real-world categories (e.g. speakers differing in gender, age, social class).

However, Samara *et al.* [12] recently obtained similar results in experimental paradigms where these factors are reduced. They report a series of three experiments where adult and child participants (age 6 years) were exposed over four sessions on consecutive days to variation in a post-nominal particle, which was conditioned (deterministically or probabilistically) on speaker identity, using the more naturalistic identity cue of speaker gender. Learners in this paradigm were tested at the end of day 1 and day 4 on their ability to produce variants matching the behaviour of both speakers in their input, or to evaluate the appropriateness of productions for each of those two speakers; the potential relevance of speaker identity was therefore highly salient from day 2 onwards. Adults and children were both able to learn that variant use was conditioned on speaker identity when that conditioning was deterministic (i.e. speaker 1 used variant 1 exclusively, speaker 2 used variant 2 exclusively); however, when variation was probabilistic (both speakers used both variants, but speaker 1 tended to produce variant 1 more often, and speaker 2 used variant 2 more often) both age groups were less successful at conditioning variant use on speaker identity: adults produced conditioned variation on day 4, but their output was less conditioned than their input; children showed sensitivity to the social conditioning in their acceptability judgements on day 4, but were unable to reproduce that conditioned usage in their own output. While further studies are required to probe more deeply into these effects, we are beginning to see converging evidence that language learners have at least some difficulty in conditioning (some types of) linguistic variation on social cues.

### (e) Discussion

Together, these experimental and computational results imply that biases for regularity in individual learners may not be enough to engender predictability in natural languages: while transmission can amplify weak biases, in some circumstances it can produce the opposite effect, masking learner biases. This means that attempting to infer learner biases from linguistic universals or predict linguistic universals from learner biases is doubly fraught: not only can strong effects in languages be due to weak biases in learners (the point we emphasized in [19]), but even very strong biases in learners can be completely invisible at the level of languages. Furthermore, the extent to which these two possibilities are true can depend on apparently unrelated features of the way learners learn (in our case, do they attend to speaker identity?), or even non-linguistic factors (how many people do learners learn from?).

## 4. Interaction

Of course, one notable feature of the models and experiments outlined in the previous section is the absence of *interaction*: learners never interact with other individuals in their generation. It seems likely, due to the well-known tendency for people to be primed by their interlocutors, i.e. to reuse recently heard words or structures, and, therefore, become more aligned (e.g. [30]), that the effects we see in multiple-person populations might be attenuated by interaction: if a learner receives input from multiple speakers who have recently interacted then their linguistic behaviour might be quite similar, which would reduce the inter-speaker variation the learner is exposed to. While this is plausible, it is worth noting that unless all speakers a learner receives input from are *perfectly* aligned and effectively functioning as a single input source, multiple-speaker effects will simply be reduced, not eliminated. Secondly, interaction may itself impact on how learners use their linguistic system, a point we turn to now.

Language is a system of communicative conventions: part of its communicative utility comes from the fact that interlocutors tacitly agree on what words and constructions mean, and deviations from the ‘usual’ way of conveying a particular idea or concept are therefore taken to signal a difference in meaning (e.g. [31,32]). This suggests that producing unpredictable linguistic variation during communication might be counter-functional—the interlocutors of a speaker who produces unpredictable variation might erroneously infer that the alternation between several forms is intended to signal *something* (i.e. is somehow conditioned on meaning). Language users might implicitly or explicitly know this, and reduce the variability of their output during communicative interaction (but might not do so in the learn-and-recall type of task we report above). Reciprocal priming between interlocutors might also serve to reduce variation: if two people interact and are primed by each other, this might automatically result in a reduction in variation (I use *fip* to mark plurality; you are more likely to use *fip* because I just did; I am more likely to use it because you just did, and so on).

There is experimental evidence to support both of these possible mechanisms. Perfors [16] trained adult participants on a miniature language exhibiting unpredictable variation in the form of (meaningless) affixes attached to object labels. While in a standard learn-and-recall condition participants reproduced this variability quite accurately, in a modified task where they were instructed to attempt to produce the same labels as another participant undergoing the same experiment at the same time (who they were unable to interact with), they produced more regular output (producing the most common affix on approximately 80% of labels, rather than on 60% as seen during training). This could be due to reduced pressure to reproduce the training language ‘correctly’ due to the changed focus on matching another participant, or it could reflect reasoning about the rational strategy to use in this semi-communicative scenario.

Other work directly tests how use of linguistic variation changes during interaction. In one recently developed experimental paradigm, participants learn a variable miniature language (either allowing multiple means of marking number or exhibiting meaningless variation in word order) and then use it to communicate ([33–35]; see also [36–38] for experimental studies looking at the role of alignment in driving convergence in graphical communication, and [39] for a review of relevant modelling work). This work finds that reciprocal priming during interaction leads to convergence within pairs of participants, typically on a system lacking unpredictable variation. Intriguingly, there is also increased regularity in participants who *thought* they were interacting with another human participant, but were in fact interacting with a computer that used the variants in their trained proportion and was not primed by the participants' productions [34]. Regularization here cannot be due to priming (as priming of the participant by the computer interlocutor should keep them highly variable), nor can it be due to reciprocal priming (as the computer is not primed by the participant); it must therefore reflect an (intentional or unintentional) strategic reduction in unpredictable variation promoted by the communicative context, consistent with the data from Perfors [16]. There are, however, subtle differences between the kind of regularization we see in this pseudo-interaction and the regularization we see in genuine interaction. Reciprocal priming in genuine interaction leads to some pairs converging on a system that entirely lacks variation, whereas pseudo-interacting participants never became entirely regular.

Finally, there are inherent asymmetries in priming that may serve to ‘lock in’ conditioned or regular systems at the expense of unpredictable variability [33,35]. While variable users can accommodate to a categorical partner by increasing their frequency of usage, categorical users tend not to accommodate to their variable partners by becoming variable: consequently, once a grammatical marker reaches a critical threshold in a population such that at least some individuals are categorical users, alignment during interaction should drive the population towards uniform categorical marker use, as variable users align to a growing group of categorical users. This, in combination with the regularizing effects of communicative task framing [16,34] and reciprocal priming [34] suggests that interaction may be a powerful mechanism for reducing unpredictable variation, which might play a role in explaining the scarcity of truly unpredictable variation in natural language.

## 5. Conclusion

The structure of languages should be influenced by biases in statistical learning, because languages persist by being repeatedly learnt, and linguistic universals may therefore reflect biases in learning. But the mapping from learning biases to language structure is not necessarily simple. Weak biases can have strong effects on language structure as they accumulate over repeated transmission. At least in some cases, the opposite can also be true: strong biases can have weak or no effects. Furthermore, learning biases are not the only pressure acting on languages: language use can produce effects that can (but need not) resemble the effects produced by learning biases, but which might have subtly or radically different causes. Combining data and insights from studies of learning, transmission and use is therefore essential if we are to understand how biases in statistical learning interact with language transmission and language use to shape the structural properties of language. We have used the learning of unpredictable variation as a test case here, but the same arguments should apply to other linguistic features: statistical learning papers frequently make inferences about the relationship between biases in statistical learning and features of language design based on studies of learning in individuals, but in the absence of a detailed understanding of how biases in learning interact with use and transmission, these inferences should be treated with caution. In our opinion, the literature on unpredictable variation provides a useful exemplar for how we should combine data from statistical learning, transmission and use in attempting to explain the universal properties of human languages.

## Endnotes

1 Similar results are obtained if we use a stronger bias in favour of regularization, e.g. setting *α* to 0.1 or 0.001—in general, we expect our results to hold as long as learners have *some* bias for regularity, but that bias is not so strong that it completely overwhelms their data.

2 See Burkett & Griffiths [23] for further discussion and results for Bayesian iterated learning in populations.

3 Because in the models and experiments we present here, all nouns occur equally frequently, we can simply calculate entropy by noun and then average (yielding the term  in the expression above)—if nouns differed in their frequency, then the by-noun entropies would be weighted proportional to their frequency, rather than a simple average. Similarly, in the expression for *H*(Marker|Noun, Speaker), we exploit the fact that all speakers are represented equally frequently in the models and experiments presented here.

4 In all conditions, to convert test output from the generation *g* participant into training input for generation *g* + 1, for a given scene, we simply inspected whether the generation *g* participant used *fip*, *tay* or no marker, and used this marking when training participant *n* + 1. In situations where the marker was mistyped, we treated it as if the participant had produced the closest marker to the typed string, based on string edit distance (e.g. ‘tip’ treated as ‘fip’). Errors in the verb or noun used were not passed on to the next participant.

5 A logit regression on the data from the two-person condition, with the presence/absence of speaker ID, shows no significant effect on majority marker use of speaker ID or the interaction between speaker ID and generation, *p* > 0.14.

6 Again, an analysis of the effect of the speaker identity manipulation in the two-person data reveals no effect on *H*(Marker) of speaker ID and no interaction between speaker ID and generation, *p* > 0.835.

7 Again, including speaker identity as a predictor indicates no effect on *H*(Marker|Noun) of speaker identity and no interaction with generation, *p* > 0.918.

8 There is weak evidence in our data that our learners are somewhat sensitive to speaker-based conditioning in their input, although clearly not sufficiently sensitive to trigger the effects predicted by the model for maximally identity-sensitive learners. For instance, there is a positive but non-significant positive correlation (*r*=0.225, *p*=0.28) between the degree of speaker-based conditioning in participants' input and their output in the two-person, speaker identity experimental data (where we measure this correlation on mutual information of marker use, noun and speaker, which is , to control for spurious correlations arising for overall levels of variability). Samara *et al.* [12], covered in the discussion, provide better data on this issue.

## Data accessibility

Raw data files for the experiment detailed in §3.4 are available at the Edinburgh DataShare repository of the University of Edinburgh: http://dx.doi.org/10.7488/ds/1462.
